# Comparative analysis of public concerns regarding treated water discharged from the Fukushima Daiichi Nuclear Power Station: perspectives before and after the initial release

**DOI:** 10.1093/jrr/rrae102

**Published:** 2025-01-11

**Authors:** Mengjie Liu, Hitomi Matsunaga, Makiko Orita, Yuya Kashiwazaki, Xu Xiao, Noboru Takamura

**Affiliations:** Department of Global Health, Medicine and Welfare, Atomic Bomb Disease Institute, Nagasaki University Graduate School of Biomedical Sciences, 1-12-4 Sakamoto, Nagasaki City, Nagasaki Prefecture 852-8523, Japan; Department of Global Health, Medicine and Welfare, Atomic Bomb Disease Institute, Nagasaki University Graduate School of Biomedical Sciences, 1-12-4 Sakamoto, Nagasaki City, Nagasaki Prefecture 852-8523, Japan; Department of Global Health, Medicine and Welfare, Atomic Bomb Disease Institute, Nagasaki University Graduate School of Biomedical Sciences, 1-12-4 Sakamoto, Nagasaki City, Nagasaki Prefecture 852-8523, Japan; Department of Global Health, Medicine and Welfare, Atomic Bomb Disease Institute, Nagasaki University Graduate School of Biomedical Sciences, 1-12-4 Sakamoto, Nagasaki City, Nagasaki Prefecture 852-8523, Japan; Department of Global Health, Medicine and Welfare, Atomic Bomb Disease Institute, Nagasaki University Graduate School of Biomedical Sciences, 1-12-4 Sakamoto, Nagasaki City, Nagasaki Prefecture 852-8523, Japan; Department of Global Health, Medicine and Welfare, Atomic Bomb Disease Institute, Nagasaki University Graduate School of Biomedical Sciences, 1-12-4 Sakamoto, Nagasaki City, Nagasaki Prefecture 852-8523, Japan

To the Editors:

The 2011 Great East Japan Earthquake and tsunami triggered a major accident at the Fukushima Daiichi Nuclear Power Plant (FDNPP), leading to significant radioactive contamination due to damaged reactor cores [1]. Since the accident, seawater has been used to cool the reactor cores, and the resulting contaminated water containing radionuclides, such as cesium-137, strontium-90 and tritium, has been collected in tanks and stored on-site at the FDNPP. To treat the contaminated water, Tokyo Electric Power Company introduced the Advanced Liquid Treatment System (ALPS), which aims to remove most of the radionuclides in the water. Although the system can reduce the levels of radioactive substances in the contaminated water to some extent, it cannot completely remove tritium [2].

To address the accumulation of ALPS treated water stored at the FDNPP, the Japanese government announced on 13 April 2021, its plan to discharge the treated water into the ocean, and the discharge officially began on 24 August 2023 [3]. Although reports (e.g. IAEA guidelines) indicate that the discharge complies with international safety standards and the environmental impact is within controllable limits [2, 3], the initiative has faced strong opposition from neighboring countries, environmental organizations and fishery communities due to concerns about potential risks to marine ecosystems and human health [4, 5]. The release of treated water from the FDNPP has thus become a complex and sensitive issue, involving considerations of environmental protection, risk communication and public trust. Therefore, it is crucial for the government and experts to understand public attitudes, particularly those of Fukushima residents and evacuees, toward the discharge of treated water in order to conduct effective risk communication and build trust. This study aims to examine the changing trends in concern regarding treated water discharge among residents and evacuees living close to the FDNPP before and after the discharge plan.

We conducted surveys in November to December 2022 (before the discharge began) and December 2023 to January 2024 (after the discharge started) in the towns of Tomioka, Okuma and Futaba, which are close to FDNPP in the Hamadori area of Fukushima Prefecture. The questionnaire used in this study was adapted from previous research and the Fukushima Health Management Survey [6, 7]. It included demographic information (e.g. age, gender and current residence). Participants were asked to assess their concerns about discharge of treated water, and their risk perception of health effects and genetic effects. Additionally, mental health status was evaluated using the mental health dimension item of the validated Japanese version of the Short Form 8 (SF-8) scale. A score of 50 ± 10 (based on the average score for the general Japanese population) on the mental health dimension indicated good mental health. Eligible participants were those aged 20 or older who could receive the questionnaire from the municipal office. Two questionnaires were distributed to each household. After excluding responses with missing data, a total of 3414 responses were included in the analysis, with 1856 responses in 2022 and 1558 in 2023.

All study protocols were reviewed and approved by the Ethics Committee of the Nagasaki University Graduate School of Biomedical Sciences (approval numbers 21082702 and 23081805). Data analysis was conducted using IBM SPSS Statistics version 28 (IBM Corp., Armonk, NY, USA), with a *P*-value <0.05 considered statistically significant.

Table 1 shows the characteristics of the participants. The proportion of respondents concerned about treated water discharge significantly decreased from 60.8% in 2022 to 41.8% in 2023 (*P* < 0.001). Regarding health and genetic risks, 44% and 49.6% of respondents, respectively, expressed concerns in 2022 dropped significantly to 33.3% and 37% in 2023 (*P* < 0.001). We conducted a binary logistic regression analysis to compare the survey results from the Hamadori area between 2022 and 2023 (Table 2). Model 1 indicated that respondents in 2023 were significantly less likely to express concerns about the discharge of treated water compared to those in 2022, with an odds ratio (OR) of 0.495 (95% CI, 0.421–0.583, *P* < 0.001). Similarly, Model 2 yielded consistent results, with an OR of 0.510 (95% CI, 0.432–0.601, *P* < 0.001).

**Table 1:** Comparison of public concerns regarding treated water discharge from FDNPP between 2022 and 2023 (chi-square test results)

Variable	Reference	Study in 2022	Study in 2023 N (%)	*P*-value
		N (%)		
Age	$<$ 60	474 (26.1)	382 (24.9)	0.43
	≥60	1340 (73.9)	1150 (75.1)	
Gender	Male	921 (50.4)	861 (55.9)	$0.001$
	Female	906 (49.6)	678 (44.1)	
Residential status	Inside Fukushima	1342 (73.4)	1122 (73.7)	0.846
	Outside Fukushima	487 (26.6)	401 (26.3)	
Concerned about discharge of treated water	Yes	1108 (60.8)	648 (41.8)	<0.001
	No	715 (39.2)	901 (58.2)	
Anxiety regarding health effects	Yes	801 (44.0)	512 (33.3)	<0.001
	No	1019 (56.0)	1024 (66.7)	
Anxiety regarding genetic effects	Yes	898 (49.6)	563 (37.0)	<0.001
	No	913 (50.4)	959 (63.0)	
Mental health status	Poor	828 (45.7)	660 (42.8)	0.092
	Good	985 (54.3)	883 (57.2)	

**Table 2:** Logistic regression analysis of public concern regarding treated water discharged from the FDNPP in 2022 and 2023

Variable	Reference	Model 1		Model 2	
		OR	95% CI	OR	95% CI
Age	<60/≥60	0.921	0.784–1.083	0.921	0.783–1.083
Gender	Male/female	1.123	0.973–1.296	1.117	0.968–1.290
Concerned about discharge of treated water	Yes/no	0.495^**^	0.421–0.583	0.510^**^	0.432–0.601
Anxiety regarding health effects	Yes/no	0.911	0.770–1.079	–	–
Anxiety regarding genetic effects	Yes/no	–	–	0.852	0.721–1.007
Mental health status	Poor/good	1.058	0.913–1.225	1.071	0.925–1.240

Reference group: 2022 survey. Note: OR; odds ratio; CI; confidence interval.

^**^ *P*<0.001.

Overall, although residents still showed relatively high levels of concern about the discharge of treated water and their perceived risks of health and genetic effects, the present results indicate a significant decrease in residents’ concerns regarding treated water discharges after the implementation of the discharge plan compared to the period before its initiation. We suggest that the reduction in concerns may be attributed to transparent communication between the government, experts and stakeholders, as well as the verification efforts by third-party organizations, such as the IAEA, which likely bolstered residents’ trust [3]. Furthermore, the socio-economic recovery in fisheries and tourism appears to have offered additional reassurance to the local population [8].

These findings emphasize the need for ongoing, transparent and science-based communications, such as regular and large-scale lectures and meetings focused on the topic of health and genetic risks and radiation safety to maintain public trust. Continuous third-party verification and open engagement with affected communities are critical to mitigating reputational impacts and ensuring lasting confidence in radiation safety. Such ongoing dialogue and third-party oversight are essential not only for supporting the socio-economic recovery of the Fukushima region but also for fostering a collaborative environment for the safe, long-term decommissioning of the FDNPP.
